# Stress Evaluation in Endodontically Treated Virtual Teeth Restored with Composite Fillings and Cast or Fiberglass Posts—A Finite Element Analysis

**DOI:** 10.3390/biomedicines13040974

**Published:** 2025-04-16

**Authors:** Mihaela-Roxana Brătoiu, Răzvan Mercuț, Monica Mihaela Iacov-Crăițoiu, Monica Scrieciu, Cătălina Măgureanu Murariu, Andreea Stănuși, Dragoș Laurențiu Popa, Veronica Mercuț

**Affiliations:** 1 Department of Prosthetic Dentistry, University of Medicine and Pharmacy of Craiova, 200349 Craiova, Romaniamonica.craitoiu@umfcv.ro (M.M.I.-C.); monica.scrieciu@umfcv.ro (M.S.); andreea.stanusi@umfcv.ro (A.S.); veronica.mercut@umfcv.ro (V.M.); 2 Department of Plastic Surgery, University of Medicine and Pharmacy of Craiova, 200349 Craiova, Romania; 3 Department of Prosthetic Dentistry, University of Medicine and Pharmacy “Carol Davila”, 032799 Bucharest, Romania; catalina.magureanu.murariu@umfcd.ro; 4 Department of Automotive, Transportation and Industrial Engineering, Faculty of Mechanics, University of Craiova, 200478 Craiova, Romania; popadragoslaurentiu@yahoo.com

**Keywords:** endodontics, post, FEM, occlusal forces

## Abstract

**Background/Objectives:** Among the complications of endodontic treatment, root fractures are the most severe and may require tooth extraction. The objective of this study was to develop virtual models of mandibular molars with different endodontic restorations to assess the stress distribution in tooth structures based on the type of corono-radicular restoration, compared with the model of an intact molar. **Methods**: Four virtual models of a mandibular molar were created: (1) an intact molar with preserved enamel, dentin, dental pulp and cementum; (2) an endodontically treated molar restored with a composite filling; (3) a molar restored with a fiberglass post and monolithic zirconia crown; (4) a molar restored with a metal cast post and monolithic zirconia crown. External force loads from 0 to 800 N were simulated using Finite Element Method (FEM). **Results**: The highest displacement, strain and stress values were observed in the molar restored with a composite filling, whereas the lowest values were recorded in the molar restored with a fiberglass post and zirconia crown. Critical stresses were primarily concentrated on the pulp chamber floor. **Conclusions**: The pulp chamber floor was identified as the most vulnerable area for fracture. This underscores the importance of preserving tooth structure to enhance the strength and durability of molars throughout and beyond endodontic treatment.

## 1. Introduction

Preventing the fracture of damaged and diseased teeth is essential for the success of endodontic treatment [1,2]. A series of studies highlight the most common complications of endodontic treatment: periapical complications [3,4], root fractures [5,6] and root resorption [7,8]. Severe fractures are often irreparable, leading to tooth loss [9]. Therefore, minimizing stress on teeth is crucial to ensuring the success of endodontic and restorative treatments aimed at preserving them.

Endodontic and restorative treatments can save damaged teeth, but the mechanical stress created by these treatments can also create dental fractures and perforations, during access cavity preparation, root canal instrumentation and irrigation, obturation methods, post space preparation, post type and coronal restoration. To minimize the stresses which cause tooth fractures, dentists must carefully select abutments for prostheses [10]. In addition, teeth must withstand mastication stresses from chewing food and proximal contacts from touching other teeth. These stresses are defined as occlusal forces and are characterized by amplitude, direction, duration and frequency. Larson et al. estimate that the amplitude of normal masticatory forces is between 9 and 180 N, with a duration of 0.25–0.33 s [11]. In young subjects, the occlusal force is between 516 and 532 N and can reach 911 N in the molar area in men with bruxism. Similar values are also reported in other studies [12,13,14] which state that in the case of functional processes, the amplitude of occlusal forces varies between 10 and 200 N, and parafunctional forces reach higher values, between 200 and 800 N. The maximum mastication forces are unevenly distributed at the level of the dental arches, with the second molar experiencing 55% of the maximum force and the incisors taking 20% of the force [11].

Tooth fracture occurs when the applied force exceeds the structural resistance [15]. Repetitive stress from bruxism, a condition characterized by involuntary clenching or grinding, can also lead to fractures [16]. Severely fractured teeth often require endodontic and restorative treatment to alleviate pain and preserve function [17].

Modern dentistry prioritizes saving damaged teeth, even when minimal residual structure remains [18]. When a crown is needed for restoration, it may require additional support from a post within the root canal [5,19,20]. Posts can be made from various materials, with selection based on factors such as biocompatibility, mechanical properties, fabrication ease and market availability.

Traditionally, cast posts have been favored for their stiffness, precise fit and high retention [21]. However, there is a growing demand for tooth-colored, non-metallic alternatives such as fiberglass posts [22]. Fiberglass posts offer elasticity similar to dentin, which may help reduce the risk of fracture and failure [5,23].

Teeth restored with posts and crowns can fracture under excessive stress. Predicting the likely fracture location, magnitude and stress distribution for different types of restorations is essential for designing restorations with a favorable prognosis [24]. Understanding stress distribution within and around the roots is crucial in addressing root fractures, a common failure in endodontically treated teeth [25].

The effect of stress on teeth can be predicted using a mathematical computer simulation known as the Finite Element Method (FEM) [11]. FEM models the impact of loading stress on restored teeth based on the materials, dental techniques and designs used [26]. By creating a 3D mathematical model, FEM simulates the geometry and loading conditions of the analyzed structure, allowing for the precise evaluation of deformation and stress distribution. It also helps identify areas of high deformation or stress concentration, providing valuable insights for optimizing dental restorations [27,28,29].

The present research is a continuation of a previous clinical study performed on endodontically treated teeth which showed that the fracture of endodontically treated teeth restored with posts occurs more frequently in teeth restored with cast posts compared with teeth restored with fiberglass posts [3].

The objective of the study was to develop four virtual models of mandibular molars, one of which was intact and three had different types of corono-radicular restorations. Based on these, the study aimed to analyze by FEM the biomechanical behavior of teeth depending on the physical and mechanical properties of the dental tissues, of the restorative materials as well as the amplitude, direction and duration of the occlusal forces.

The secondary objectives of this study were to determine the moment when the stresses on endodontically treated teeth become critical.

The null hypothesis assumed that there is no difference in stress distribution depending on the amplitude, direction and duration of occlusal forces in teeth restored with different combinations of corono-radicular materials.

## 2. Materials and Methods

The present study is an “in silico” study and is based on an “in vivo” model of a mandibular molar.

### 2.1. Human Tooth

The collection and use of an extracted human tooth for this study was approved by the Ethics Committee of the University of Medicine and Pharmacy Craiova (No. 212/10.11.2022).

For the present study, a mandibular molar with a carious lesion was used, for which, in a previous study conducted using FEM, the highest stress values were obtained depending on the shape of the access cavity and the direct restoration material (composite and amalgam) [30]. Because our previous clinical study [3] showed that 73.9% of root fractures occurred in teeth restored with crown fillings, 8.69% of fractures occurred in teeth with cast posts, and 4.34% of fractures occurred in teeth restored with fiberglass posts, for the present study, we chose these types of restorations. Fractures originating from reconstructed teeth with screwed posts were not considered because the pressure exerted during screwing cannot be quantified using FEM.

### 2.2. Virtual Tooth Models

To achieve the objectives of the study, four virtual models of the molar were created. Thus, the following situations were simulated:

- Model no. 1: Molar without endodontic treatment, containing enamel, dentin, pulp and cementum;
- Model no. 2: Molar after endodontic treatment, containing enamel, dentin and cementum, with a gutta-percha root canal filling and composite filling;
- Model no. 3: Molar after endodontic treatment, containing enamel, dentin and cementum, with gutta-percha root canal filling, restored with fiberglass post, composite filling and monolithic zirconia crown;
- Model no. 4: Molar after endodontic treatment, containing enamel, dentin and cementum, with gutta-percha root canal filling, restored with a chromium–nickel alloy post and monolithic zirconia crown.

**Figure 1:**
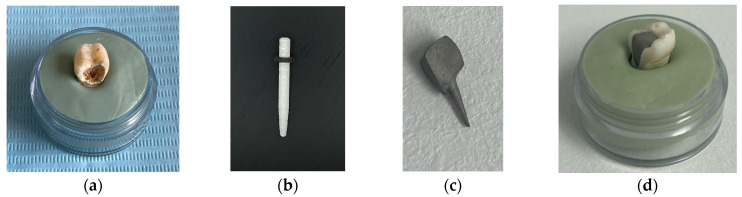
(**a**) The molar used in the study; (**b**) fiber post; (**c**) chromium–nickel alloy post; (**d**) the molar with the chromium–nickel alloy post.

The tooth used was a mandibular molar shown in Figure 1a. For the creation of Model no. 1 and Model no. 2, data from the previous study were taken [30]. For the creation of Model no. 3 and Model no. 4, the tooth was prepared for a metal cast post. A chromium–nickel alloy post was cast for the given situation and a fiberglass post was also adapted, from which the simulation in the FEM analysis started, both for the cast post and for the fiberglass post (Figure 1).

### 2.3. Hardware, Software and Appliances

**Figure 2:**
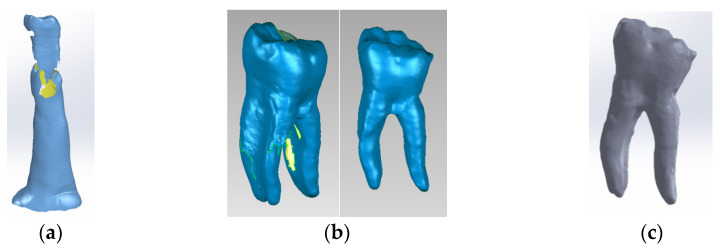
(**a**) Stages of scanning the external model of the studied molar, colored in blue (with silicone support)- the yellow color marks the unscanned area; (**b**) stages of processing the external model of the studied molar, colored in blue (after the silicone support structure was eliminated)- yellow color marks the unscanned area; (**c**) the final model loaded into the SolidWorks program, colored in grey.

The nickel–chromium alloy post and the four virtual models of the human molar were created using a 3D Systems Capture scanner (Rock Hill, SC, USA) (Figure 2), with two desktop computers running. Geomagic for SolidWorks program, version 2015 (3D Systems, Rock Hill, SC, USA), was used to determine the primary geometries [31]. The four virtual models were transformed into virtual solids, using Computer-Aided Design (CAD) (SolidWorks 2021, Ansys Workbench 2019, Dassault Systèmes, Velizy-Villacoublay, France) [32].

The FEM mechanical stress simulations on the four models of virtual teeth were analyzed using Ansys Workbench (Ansys, Inc., Canonsburg, PA, USA) [33,34]. The data and results were graphed using Microsoft Office (Microsoft Corporation, Redmont, OR, USA). FEM models were used to investigate the mechanical behavior of the virtual teeth [30] following endodontic treatment and restoration.

### 2.4. The Creation of the Virtual Molars

The following methods were used to obtain virtual models:

- Methods and principles of Continuum Mechanics applied to operating with Multi Body virtual solids;
- Techniques and methods specific to the strength of the materials applied to defining loading systems and modeling mechanical constraints;
- Methods and principles of Computer-Aided Design (CAD) and Direct Engineering and Virtual Prototyping applied to modeling the composite and posts;
- Methods and techniques of reverse engineering applied to the three-dimensional scanning of the analyzed molar and the metal post;
- Techniques and methods specific to the Finite Elements Method (FEM) applied to the studied models to determine the mechanical behavior [30];
- Principles and techniques of restoration of teeth with endodontic treatment [35,36].

Model no. 1 was created using the 3D Systems scanner while embedded in a silicone support (Figure 2a). The support was digitally removed (Figure 2b) to reveal the final virtual form, loaded into the SolidWorks program (Figure 2c).

**Figure 3:**
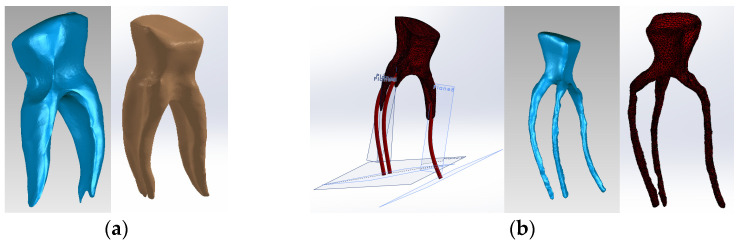
(**a**) Three-dimensional model of the dentin of the studied molar; (**b**) three-dimensional model of the pulp of the studied molar.

In the Geomagic program, the internal structures of the virtual tooth were modeled to represent dentin (Figure 3a), and the dental pulp (Figure 3b). Finally, the model was loaded into SolidWorks and transformed into a virtual solid.

**Figure 4:**
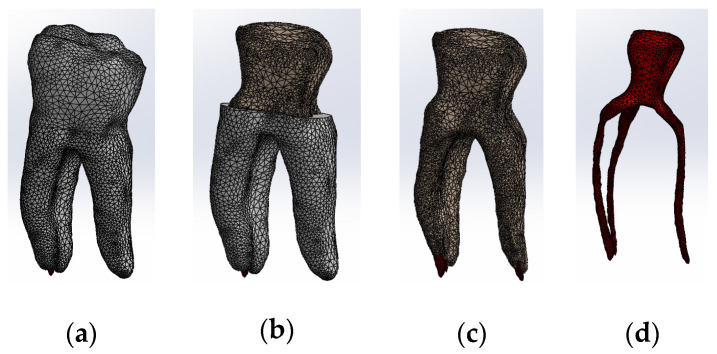
Three-dimensional Model no. 1—Working stages in Assembly module of SolidWorks: (**a**) initial complete model; (**b**) after enamel volumetric subtraction; (**c**) after cementum volumetric subtraction; (**d**) after dentin volumetric subtraction (the pulp is colored dark red).

The three models (external, dentin and pulp models) were loaded into the Assembly module of SolidWorks and aligned based on the common coordinate system of the enamel, dentin and pulp models [30,37,38,39]. Volumetric subtraction techniques were applied for enamel and dentin. Also, the specific volumes of enamel and dental cementum were defined (Figure 4).

**Figure 5:**
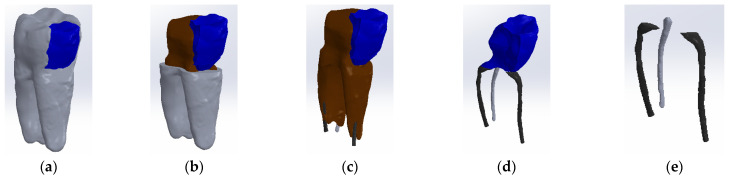
Three-dimensional Model no. 2—Working stages in Assembly module of SolidWorks: (**a**) initial complete model (enamel and cementum are colored grey, composite is colored blue); (**b**) after enamel volumetric subtraction (dentin is colored brown); (**c**) after cementum volumetric subtraction; (**d**) after dentin volumetric subtraction; (**e**) after composite volumetric subtraction.

To obtain Model no. 2, techniques and methods specific to forward and reverse engineering were applied. Thus, the model in Figure 5 was obtained, where the composite is colored blue.

**Figure 6:**
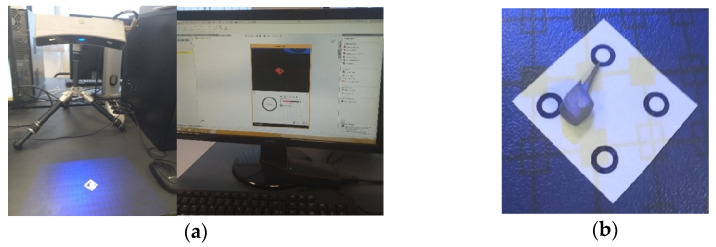
(**a**) Three-dimensional scanning system; (**b**) scanning markers and cast post.

To obtain Model no. 3 and Model no. 4, 3D scanning techniques were used, using the 3DSYSTEMS CAPTURE 3D scanner (Scanner 3D Systems, Rock Hill, SC, USA). The scanning system was composed of a desktop computer with an INTEL Core I3 processor and the Geomagic for SolidWorks program (Figure 6a). For easier alignment of scanned surfaces, scanning markers were used as shown in Figure 6b.

**Figure 7:**
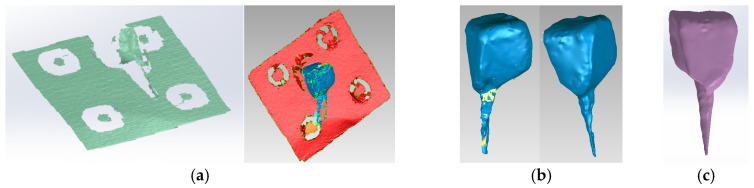
(**a**) Scanning, alignment and processing operations of scanned post (scanning markers are colored green and then red, the post is blue); (**b**) reverse engineering operations applied to the scanned post, colored blue (the yellow color marks the unscanned area); (**c**) final model of the cast post in Geomagic and SolidWorks, colored magenta.

The virtual posts were generated by scanning an actual cast post (Figure 6b). The scans were then aligned to create a 3D virtual solid model (Figure 7a,b). Figure 7c shows the final model of the cast post in Geomagic and in SolidWorks, where it was transformed into a virtual solid.

**Figure 8:**
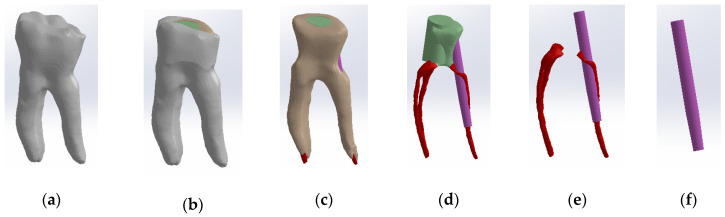
Three-dimensional Model no. 3—Working stages in Assembly module of SolidWorks: (**a**) initial complete model; (**b**) after enamel volumetric subtraction; (**c**) after cementum volumetric subtraction; (**d**) after dentin volumetric subtraction (composite part of the reconstruction is colored green, gutta-percha is colored red, fiberpost is colored magenta); (**e**) after composite volumetric subtraction; (**f**) fiberglass post.

To obtain Model no. 3, the shape of the scanned cast post was also used to adapt the fiberglass post. The core part was considered the composite part of the reconstruction (green colored) and the post part, the actual fiberglass post (magenta colored) (Figure 8). To differentiate the stresses due to the properties of the restorative materials, the final shape of the monolithic zirconia crown was identical to the shape of the tooth restored by filling.

**Figure 9:**
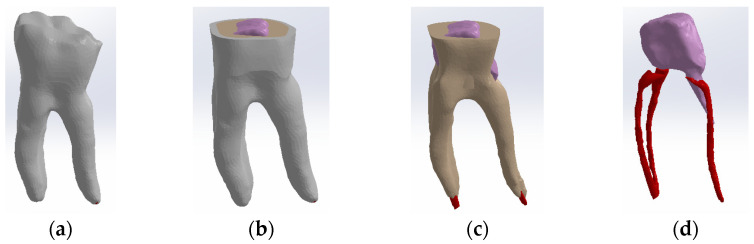
Three-dimensional Model no. 4—Working stages in Assembly module of SolidWorks: (**a**) initial complete model; (**b**) after enamel volumetric subtraction; (**c**) after cementum volumetric subtraction (dentin is colored brown); (**d**) after dentin volumetric subtraction (cast post is colored magenta, gutta-percha is colored red).

To obtain Model no. 4, the scans were loaded into the Assembly module of SolidWorks, where the post is magenta and the gutta-percha is red (Figure 9).

### 2.5. Assignation of Properties of the Materials Used to the Virtual Models

The tooth structures and dental materials analyzed in this study had distinct densities, moduli of elasticity and Poisson’s ratios, as presented in Table 1.

**Figure 10:**
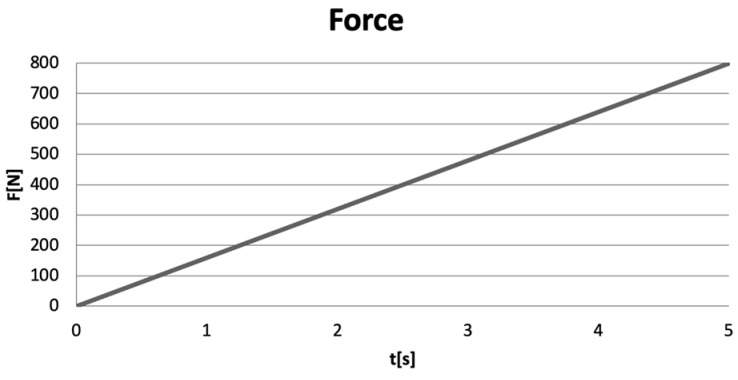
Force variation graph in Ansys Workbench.

Excessive occlusal forces were applied to the models of virtual teeth, forces ranging from 0 to 800 N, over a duration of 5 s [12,13,14]. A force variation graph illustrating the linear application of the forces (Ansys Workbench) is shown in Figure 10.

**Figure 11:**
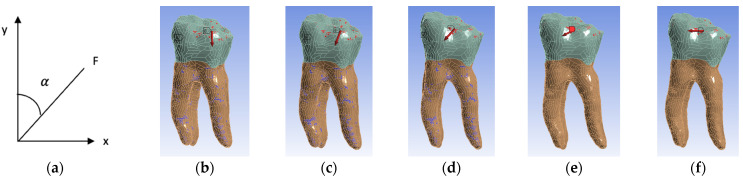
(**a**) Angle measurement; (**b**) 0 ° angle inclination (vertical); (**c**) 22.5° angle inclination; (**d**) 45° angle inclination; (**e**) 67.5° angle inclination; (**f**) 90° angle inclination (horizontal). For all figures, the coronal area is colored green and the radicular area is colored brown. A marks the surfaces that were considered fixed, and B the surfaces on which the force acts.

Five simulation scenarios were simulated depending on the direction of action of the occlusal forces. In the first scenario, the forces had a vertical direction corresponding to an angle of 0°, three scenarios with oblique directions at 22.5°, 45° and 67.5° and the last scenario with a horizontal direction at an angle of 90 ° (Figure 11). The angle (α) between the long axis of the tooth (y) and the applied force (F) was measured (Figure 11a).

The stress analysis of the virtual teeth using FEM included the following parameters:

- Displacement state provides information on the variation in node positions of the finite elements, expressed in meters (m);
- Strain state was evaluated using von Mises criterion, representing the elongation of finite elements relative to the unit of length, expressed in meters per meter (m/m);
- Stress state was determined using von Mises algorithm which quantifies the loading of finite elements by relating force to surface, expressed in Pascals (1 Pa = 1 N/m^2^).

### 2.6. Statistical Analysis

Statistical tests were performed using Statistical Package for Social Sciences (SPSS), version 26 (IBM Corp., Armonk, NY, USA). The descriptive analysis reflects continuous variables expressed as mean ± standard deviation. Statistical analysis included Shapiro–Wilk’s test for data normality analysis, Levene’s test of equality of variances and one-way ANOVA for group comparisons. For the present study, the value *p* < 0.05 was considered statistically significant.

## 3. Results

### 3.1. Simulation of the Action of Excessive Occlusal Forces

**Figure 12:**
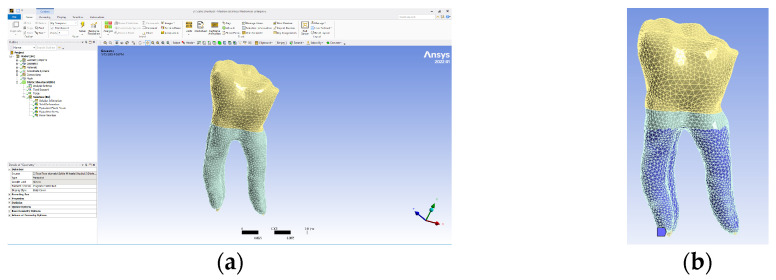
(**a**) Ansys Workbench program interface and Model no. 1; (**b**) the fixed surfaces of the analyzed assembly (in shades of blue).

The model obtained in the Assembly module of SolidWorks was imported into Ansys Workbench, and the interface of this program is presented in Figure 12a. As a rule, it was established that the part of the teeth that is normally fixed in the alveolar bone was colored in blue (Figure 12b).

**Figure 13:**
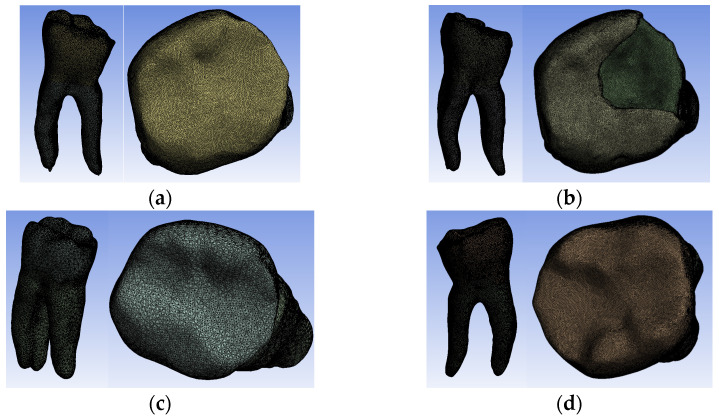
Finite element structure of: (**a**) Model no. 1; (**b**) Model no. 2; (**c**) Model no. 3; (**d**) Model no. 4.

All four models were divided into finite elements, resulting in the following:

- Model no. 1: 5,621,752 nodes and 3,780,531 elements (Figure 13a);
- Model no. 2: 717,841 nodes and 3,542,032 elements (Figure 13b);
- Model no. 3: 3,019,940 nodes and 1,701,336 elements (Figure 13c);
- Model no. 4: 910,776 nodes and 4,347,626 elements (Figure 13d).

### 3.2. Numerical Results Obtained from the Simulation

The numerical results were obtained based on the distribution maps of displacements, strains and stresses. Since horizontal occlusal forces are considered the most harmful, the biomechanical behavior of the four virtual models under the action of these forces will be exemplified.

#### 3.2.1. Numerical Results for Model No. 1

**Figure 14:**
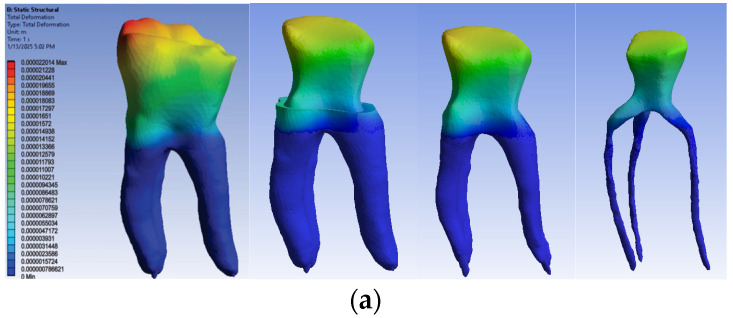

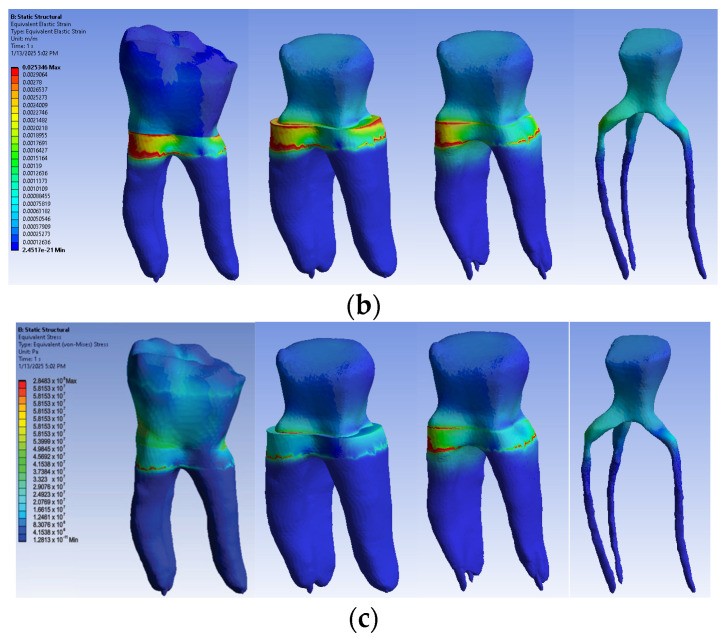
Model no. 1 maps for (**a**) displacements; (**b**) strains; (**c**) stresses.

The simulation results consist of displacement, strain and stress maps. The displacement, strain and stress maps of Model no. 1 are presented in Figure 14. To be able to observe the internal dental structure, the enamel, cementum and dentin, models were temporarily and successively suppressed, as shown in Figure 14 from left to right. The areas with the highest values of displacements, strains and stresses developed inside the structures are colored in red and are primarily located in the pericervical area.

#### 3.2.2. Numerical Results for Model No. 2

**Figure 15:**
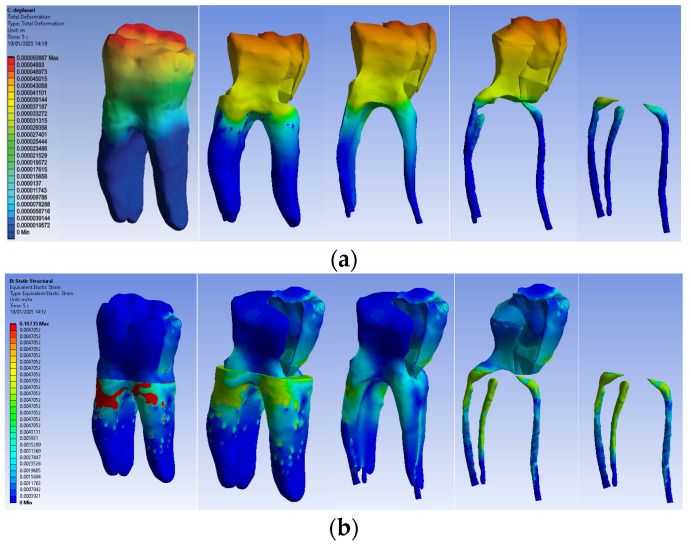

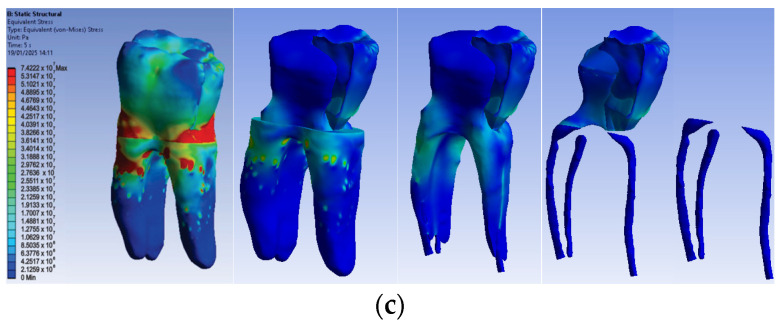
Model no. 2 maps for (**a**) displacements; (**b**) strains; (**c**) stresses.

The simulation results consist of displacement, strain and stress maps. The displacement, strain and stress maps of Model no. 2 are presented in Figure 15. To analyze the internal tooth structure, as well as the enamel, cementum, dentin and composite material, models were temporarily and successively suppressed, as shown in Figure 15 from left to right. The areas with the highest values of displacements, strains and stresses developed inside the structures are colored in red and are primarily located in the pericervical area, but more extended in the root area.

#### 3.2.3. Numerical Results for Model No. 3

**Figure 16:**
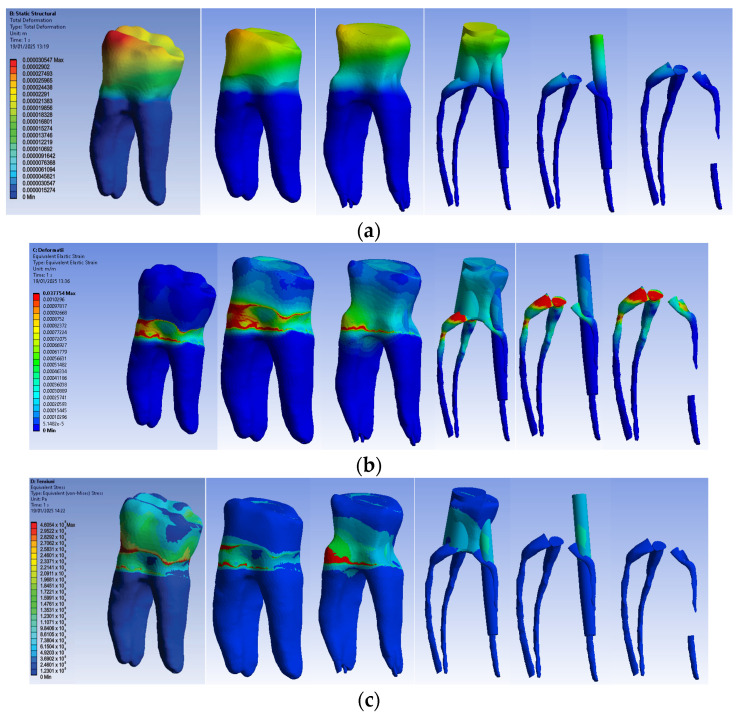
Model no. 3 maps for (**a**) displacements; (**b**) strains; (**c**) stresses.

The simulation results consist of displacement, strain and stress maps. Figure 16 shows the displacement, strain and stress maps of Model no. 3. To analyze the internal tooth structure, the crown, cementum, dentin, composite material and fiberglass post, models were temporarily and successively suppressed, as shown in Figure 16 from left to right. The areas with the highest values of displacements, strains and stresses developed inside the structures are colored in red and are primarily located in the pericervical area but more extended in the conoral area and the pulp chamber floor.

#### 3.2.4. Numerical Results for Model No. 4

**Figure 17:**
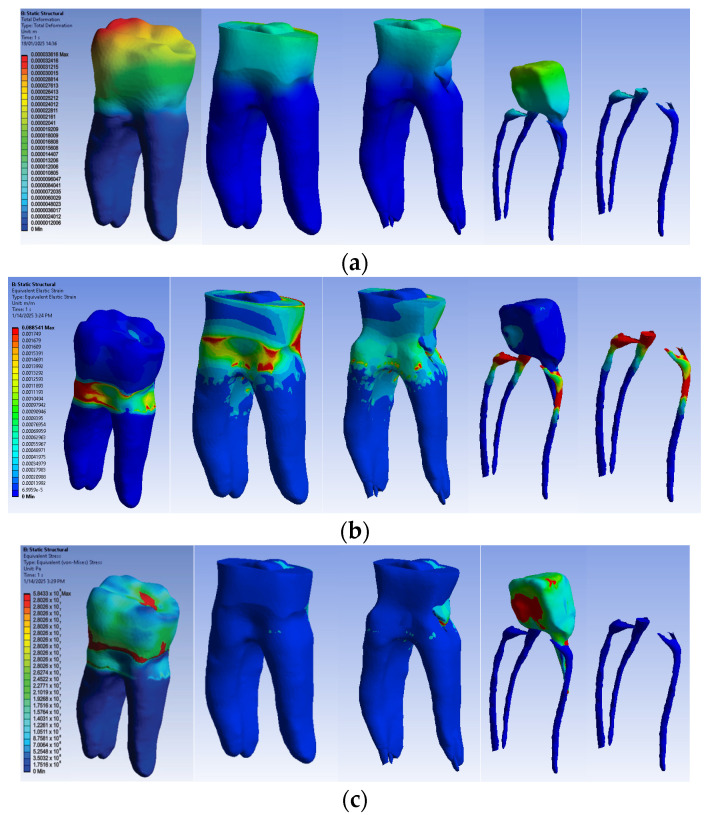
Model no. 4 maps for (**a**) displacements; (**b**) strains; (**c**) stresses.

The simulation results consist of displacement, strain and stress maps. Figure 17 shows the displacement maps of the Model no. 4. To analyze the internal tooth structure, the crown, cementum, dentin, composite material and metal cast post, models were temporarily and successively suppressed, as shown in Figure 17 from left to right. The areas with the highest values of displacements, strains and stresses developed inside the structures are colored in red and are primarily located in the pericervical area, the cast area and the pulp chamber floor.

### 3.3. Comparison of Displacements, Strains and Stresses Obtained in the Simulations of the Action of Horizontal Forces

**Figure 18:**
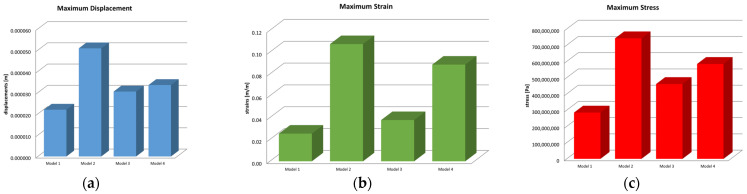
Comparative diagram of (**a**) maximum displacements; (**b**) maximum strains; (**c**) maximum stress.

Microsoft Office package was used to obtain graphs and diagrams, but also to organize the data extracted from the result maps. Thus, a comparative diagram was defined for the maximum displacements, strains and stresses, resulting from the four situations analyzed under the action of the excessive horizontal forces, and the diagrams are presented in Figure 18.

Centralizing the information from the three diagrams, the results are presented in the data in Table 2, which represent the maximum values recorded for the four models, specifying that their distribution at the level of the virtual model components is not uniform. Stress values have been converted to megapascals (MPa) for easier comparison with reference values.

### 3.4. Statistical Analysis of Stress in Virtual Models

**Figure 19:**
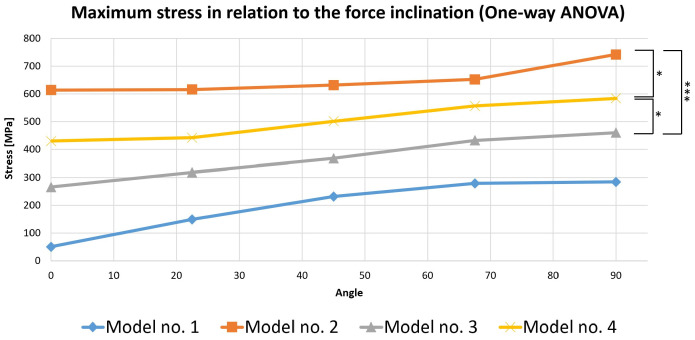
Comparative chart of maximum stress depending on force inclination (the number of asterisks defines the following statistical thresholds: <0.05 (*), <0.001 (***)).

To perform this evaluation, five occlusal loading scenarios were considered depending on the direction of the force on the selected virtual models. These models were represented by the three mandibular molars with corono-radicular restoration. Since stress is the most important variable analyzed in the previous simulations, the calculations were made based on it (Figure 19).

A one-way ANOVA was conducted to determine if the stress was different for the three models with restorations. There were no outliers, as assessed by a boxplot; data were normally distributed for each group, as assessed by the Shapiro–Wilk test (*p* > 0.05); and there was a homogeneity of variances, as assessed by Levene’s test of homogeneity of variances (*p* = 0.535). The stress was statistically significantly different for the three groups, F(2, 12) = 21.621, *p* < 0.0005. The mean values of stress increased from Model 3 (369.444 ± 79.773) to Model 4 (503.130 ± 67.955), and then to Model 2 (651.402 ± 53.095), in that order. Tukey’s post hoc analysis revealed that the increase from Model 3 to Model 4 (133.686, 95% CI (19.243 to 248.128)) was statistically significant (*p* = 0.022), as was the increase from Model 3 to Model 2 (281.958, 95% CI (167.515 to 396.400), *p* < 0.0005) and the increase from Model 4 to Model 2 (148.272, 95% CI (33.829 to 262.714), *p* = 0.012).

### 3.5. Determining When the Force Becomes Critical

In order to analyze the impact of stress developed during simulations, the data obtained in the study were compared with the fracture resistance referred in the specialized literature as “flexural strength” [46].

The time (t) and force (F) when the reference value was exceeded were analyzed. The values that exceeded the reference (considered critical) are marked in the following table using a bold format of writing (Table 3).

For Model 3 and Model 4, the critical values were not reached during the simulation due to the increased strength above the critical threshold of the zirconia crown. The table shows the maximum values recorded during the simulations.

The null hypothesis was not confirmed, due to there being a difference in stress distribution depending on the amplitude, direction and time of action of the occlusal forces in teeth restored with different combinations of corono-radicular materials.

## 4. Discussion

The present study conducted using FEM addresses a particularly important aspect for practitioners in the fields of dentistry, prosthetics and endodontics, namely the therapeutic conduct that must be adopted after endodontic treatment, to integrate the respective tooth into the morpho-functionality of the dento-maxilarry apparatus (DMA) and to ensure a good prognosis over time. The study evaluated the behavior of three virtual models of endodontically treated molars, restored by the most common current restoration methods, compared to an intact model of the molar.

The results of the present study confirmed the results obtained in the previous clinical study mentioned in the introduction, which refers to the evolution of 548 teeth with endodontic treatment, restored by various methods [3]. According to the specified clinical study, from which the current research started, the teeth with endodontic treatment most susceptible to fracture are those restored by coronal fillings, followed by teeth restored with cast posts and teeth restored with fiberglass posts.

Several important aspects must be considered when determining the way to restore teeth with endodontic treatment, including the amount of remaining dental tissues and the stresses to which the tooth will be subjected [47,48].

A series of “in silico” studies conducted using FEM [49,50,51] and “in vitro” studies conducted through laboratory simulation [52,53] have analyzed the behavior of teeth restored by various methods.

The main reasons for endodontic treatment are represented by advanced dental caries and dental trauma that cause the loss of a significant amount of dental tissues. Added to these are the design of the access cavity and the instrumentation of the root canal, all of which cause a decrease in the strength of the teeth with endodontic treatment. Endodontic procedures are considered to reduce tooth stiffness by 5%, by creating the access cavity, while the removal of tooth structure in a mesio-occlusal-distal (MOD) cavity reduces tooth stiffness by 60% [9,54]. The properties of dentin are irreversibly modified by endodontic procedures due to collagen degradation and dentin dehydration, resulting in a 14% reduction in the strength of endodontically treated molars [9]. The main objectives of the restoration of endodontically treated teeth are to maintain the integrity and function of the teeth and to achieve a satisfactory aesthetic result [48]. In addition to the forces to which endodontically treated teeth are subjected during functional processes [55] and the excessive ones from bruxism or other unfavorable situations related to dental occlusion, it should be noted that endodontically treated teeth included in fixed or removable prosthetic restorations have a poorer prognosis than vital teeth [48,56,57,58,59]. However, not every endodontically treated tooth requires extensive restoration. The decision for the restoration method is made depending on the topography of the tooth on the arch, the amount of remaining tissues, the size and morphology of the root and the demands to which the tooth will be subjected [9,60]. When a crown is required to successfully restore a tooth, it must be determined whether a post and core is necessary. A post and core will be necessary if there is insufficient tooth structure remaining to retain a crown or if the dentin is so weak that a crown fracture is likely to occur [61]. This is a subjective decision and will depend on the amount and location of the remaining tooth structure, as well as the type of crown to be made [48]. Studies support that a post and core do not augment the remaining tooth, and its primary role is to provide retention of the crown restoration [62]. It is useful to first prepare the tooth for the crown and then assess the amount of remaining dentin. It is difficult to judge how much remaining dentin is acceptable. The results of a review showed that the presence of a 1.5–2 mm “ferrule” has a positive effect in ensuring an adequate form of resistance for a tooth [63] and in preventing root fracture [36,64].

A previous study also conducted using FEM [30] showed that, regardless of the design of access cavities, the pericervical dentin experienced the highest level of stress. The same result was obtained in this study; the highest level of displacements, strains and stress was in the same pericervical area, proving the importance of preserving pericervical dentin.

For the present study, four virtual models of molars were created: the first model was of an intact molar, used as a control sample, in order to be able to refer to it; the next model was the one with direct composite restoration, which is the easiest to achieve practically in the restoration of teeth with endodontic treatment; one model was restored with a fiberglass post and zirconia crown; and the final model was restored with cast metal post and zirconia crown. The latter two represent the most common clinical modalities for restoring teeth with endodontic treatment. Zirconia restoration was selected because its mechanical properties provide excellent mechanical strength in dental applications.

Although there is a wide variability of materials, their mechanical properties do not coincide with those of dental tissues (modulus of elasticity: enamel 8.41 × 1010 Pa, dentin 1.86 × 1010 Pa, fiberglass post 4 × 1010 Pa [65], chromium–nickel alloy 2.1 × 1011 Pa [45], zirconia 2.05 × 1011 Pa [66].

Fiberglass posts have properties similar to those of dental tissue (dentin) [9]. In structurally intact teeth, non-rigid posts flex with the tooth under functional forces, thereby reducing force transfer to the root and reducing the risk of root fracture. In teeth with significant tissue loss, which lack the cervical rigidity of dentin, excessive post-flexion can be detrimental to marginal closure [9,60].

Cast metal posts have been the standard in the restoration of teeth with significant tissue loss, having been widely used in the past with good results [9]. They are rigid, have the advantage of good adaptation to the remaining tissues and can be removed from the tooth in case of reinfection of the root canal, but they require the involvement of the laboratory for fabrication [9].

A series of studies have compared the effects of the two types of posts in terms of the evolution of restored teeth [67,68,69]. Thus, the results showed that the use of metal posts has a more harmful effect than fiber posts, because the modulus of elasticity of fiber posts is comparable to that of dentin [70]. In an article by Sethuraman in 2011, the results showed that fiberglass posts presented the most favorable stress distribution, similar to the structure of the natural tooth [71].

The results of the present study overlapped with these findings. Pegoretti et al. used FEM in a study and observed that teeth restored with fiberglass posts showed a lower stress concentration within the root compared to teeth restored with metal and carbon fiber posts [72]. The greater the difference between Young’s modulus of the dentin and posts, the less homogeneous the stress distribution on the tooth surface is, thus producing areas of stress concentration on the dentin [28]. The behavior of a fiber-restored tooth was similar to that of a natural tooth, as it produces a homogeneous stress distribution. In a review by Badami in 2022, the results showed that fiberglass posts induce a more homogeneous stress distribution, with the least stress on the restored teeth compared to other posts [73]. The results also showed that the stress distribution in teeth restored with fiberglass posts, although homogeneous, is mostly concentrated in cervical regions, while in teeth with stainless steel, titanium, zirconia and cast posts, stress was present in the post, cervical and apical regions of the tooth structure. Therefore, fiber post fractures are less likely to occur in the root [74].

In a 2020 review by Nahar et al., out of 22 studies, 15 studies evaluated fiberglass posts, and the results showed that they induce less stress on endodontically treated restored teeth compared to cast posts, with stress concentration within the post, cervical and apical regions of the tooth structure [23]. Another study conducted in 2021 found that failure rates were similar for restorations with fiber posts and metal posts in endodontically treated teeth [75]. In vivo and in vitro studies have demonstrated that fiberglass posts combined with a composite resin build-up are an excellent approach for the restoration of endodontically treated teeth compared to cast or prefabricated metal posts, resulting in excellent clinical performance [5,76].

The weakening of the tooth’s mechanical strength increases the risk of a vertical root crack or even a fracture, which clinically means its loss is one of the most serious complications of a tooth reconstructed with posts [59]. This is why the current therapeutic protocol recommends complete, adequate crown protection as a final preventive procedure after conservative orthograde endodontic treatment [77].

To prove the role of excessive forces in the evolution of endodontically treated restored teeth, the simulation was performed based on occlusal forces whose amplitude increased from 0 to 800 N, their direction of action varied from 0 to 90° and the duration of action was between 0 and 5 s.

Other studies conducted using FEM have also evaluated the biomechanical behavior of teeth depending on the amplitude and direction of occlusal forces [78]. The results obtained in this study overlap with their results.

Comparing the maximum values obtained in the study with the flexural strength values of each material/tissue included in the study, it was found that for Models 1 and 2 the critical values were exceeded, while in Models 3 and 4, due to the zirconium crown properties, these values were not exceeded.

We did not find any data on the flexural strength of cementum in the literature, and the explanation could be that the mineralization of cementum and changes in its width do not stop with initial formation but continue over time [79]. Also, the cementum is not directly fused to dentin. It is attached to dentin via CDJ (cementum dentin junction) and to the periodontal ligament via PCE (periodontal ligament-cementum enthesis) [80].

The limitations of the study are that the cement and composite films used for cementing the posts were excluded from the study due to their reduced thickness. Also, the study could not fully reproduce real conditions, such as the physiological tooth mobility which appears due to periodontal ligaments.

## 5. Conclusions

Using 3D modeling and numerical analysis, it was possible to understand how the selection of different restoration materials affects stress distribution.

The behavior of teeth with endodontic treatment depends on the corono-radicular restoration as well as the stresses to which it will be exposed, reflected in this study through the amplitude, direction and duration of action of occlusal forces.

The pulp chamber floor was identified as the most vulnerable area for fractures. This underscores the importance of preserving tooth structure to enhance the strength and durability of molars throughout and beyond endodontic treatment.

The study has practical applicability and can guide practitioners in choosing the right therapeutic approach for teeth with endodontic treatment.

## Figures and Tables

**Table 1:** Physical and mechanical properties of materials used in finite element analysis.

Material	Density [kg/m^3^]	Modulus of Elasticity E [Pa]	Poisson’s Ratio
Enamel	2958	8.41 × 10^10^	0.3 [40]
Dentin	2140	1.86 × 10^10^	0.31 [40]
Dental pulp	1000	2 × 10^6^	0.45 [40]
Cementum	2063	1.55 × 10^9^	0.31 [41,42]
Gutta-percha	1100	6.9 × 10^6^	0.45 [40]
Composite	1151.9	1.277 × 10^10^	0.31 [40]
Fiberglass post	2000	4 × 10^10^	0.26 [43]
Zirconia	4650	2.05 × 10^11^	0.22 [44]
Chromium–nickel alloy	8500	2.1 × 10^11^	0.31 [45]

**Table 2:** Maximum displacements, strains and stresses.

	Model No. 1	Model No. 2	Model No. 3	Model No. 4
Displacements [m]	0.00002201	0.000050887	0.000030547	0.000033614
Strains [m/m]	0.025346	0.10735	0.037754	0.088541
Stress [MPa]	284.83	742.22	460.54	584.33

**Table 3:** Comparison of the maximum values obtained in simulations with a reference value of flexural strength of each material/tissue.

Material/Tissue	Values Obtained	Unit of Measure	Model No. 1	Model No. 2	Model No. 3	Model No. 4
Enamel	Reference value **179 MPa [46]**	t(s)	**3.63**	**3.77**	-	-
		F(N)	**581.94**	**604.11**	-	-
	Maximum values obtained	t(s)	5	5	-	-
		F(N)	800	800	-	-
		MPa	**246.07**	**237.04**	-	-
Dentin	Reference value **212 MPa [46]**	t(s)	**3.77**	**2.89**		
		F(N)	**604.11**	**462.90**		
	Maximum values obtained	t(s)	5	5	5	5
		F(N)	800	800	800	800
		MPa	**284.83**	**375.02**	63.48	158.81
Composite	Reference value **150 MPa [46]**	t(s)	-	-		
		F(N)	-	-		
	Maximum values obtained	t(s)	-	5	5	5
		F(N)	-	800	800	800
		MPa	-	114.46	3.08	-
Fiberglass post	Reference value **879 MPa [46]**	t(s)	-	-		
		F(N)	-	-		
	Maximum values obtained	t(s)	-	-	5	5
		F(N)	-	-	800	800
		MPa	-	-	1.99	-
Cast post (Cr-Ni alloy)	Reference value **900 MPa [46]**	t(s)	-	-		
		F(N)	-	-		
	Maximum values obtained	t(s)	-	-	5	5
		F(N)	-	-	800	800
		MPa	-	-	-	82.64
Zirconia crown	Reference value **1200 MPa [46]**	t(s)	-	-		
		F(N)	-	-		
	Maximum values obtained	t(s)	-	-	5	5
		F(N)	-	-	800	800
		MPa	-	-	460.54	584.33

## Data Availability

The authors declare that the data of this research are available from the corresponding authors upon reasonable request.

## Abbreviations

The following abbreviations are used in this manuscript:

DMA	Dento-maxillary apparatus
FEM	Finite Element Method
3D	Three-dimensional

## References

[1] López-Valverde I., Vignoletti F., Vignoletti G., Martin C., Sanz M. (2023). Long-term tooth survival and success following primary root canal treatment: A 5- to 37-year retrospective observation. Clin. Oral Investig..

[2] Fransson H., Dawson V. (2023). Tooth survival after endodontic treatment. Int. Endod. J..

[3] Boțilă M.R., Țuculină M.J., Diaconu O.A., Ionescu M., Mărășescu P.C., Lascu L.C., Mercuț V. (2024). Retrospective study of the evolution of teeth with endodontic treatment in a group of patients from Craiova- România. Rom. J. Oral Rehabil..

[4] Gheorghe A.G., Mercuț V., Popescu S.M., Banica A.C., Ionescu M., Gheorghiță L.M., Moraru A. (2019). Frequency of endodontic treatment and prevalence of apical periodontitis in abutment teeth—A radiological study. Rom. J. Oral Rehabil..

[5] Moris I.C.M., Moscardini C.A., Moura L.K.B., Silva-Sousa Y.T.C., Gomes E.A. (2017). Evaluation of Stress Distribution in Endodontically Weakened Teeth Restored with Different Crown Materials: 3D-FEA Analysis. Braz. Dent. J..

[6] Monga P., Sharma V., Kumar S. (2009). Comparison of fracture resistance of endodontically treated teeth using different coronal restorative materials: An in vitro study. J. Conserv. Dent..

[7] Bănică A.C., Marinescu I.R., Gheorghe D.N., Trușcă A.G., Drăghici E.C., Mercuț V., Popescu S.M. (2018). Root resorbtion prevalence in adults from Dolj county, Romania- A radiological evidence. Rom. J. Oral Rehabil..

[8] Chieruzzi M., Pagano S., De Carolis C., Eramo S., Kenny J.M. (2015). Scanning Electron Microscopy Evaluation of Dental Root Resorption Associated with Granuloma. Microsc. Microanal..

[9] Kalra H., Sukhija U., Rassawet R., Rani V. (2020). A Review on Post and Core. Sch. J. Dent. Sci..

[10] Tang W., Wu Y., Smales R.J. (2010). Identifying and reducing risks for potential fractures in endodontically treated teeth. J. Endod..

[11] Larson T.D. (2014). The effect of occlusal forces on restorations. J. Mich. Dent. Assoc..

[12] Sagl B., Schmid-Schwap M., Piehslinger E., Kundi M., Stavness I. (2022). Effect of facet inclination and location on TMJ loading during bruxism: An in-silico study. J. Adv. Res..

[13] Sagl B., Schmid-Schwap M., Piehslinger E., Rausch-Fan X., Stavness I. (2022). The effect of tooth cusp morphology and grinding direction on TMJ loading during bruxism. Front. Physiol..

[14] Sarıkaya I., Hayran Y. (2018). Effects of dynamic aging on the wear and fracture strength of monolithic zirconia restorations. BMC Oral Health.

[15] Fan J., Caton J.G. (2018). Occlusal trauma and excessive occlusal forces: Narrative review, case definitions, and diagnostic considerations. J. Periodontol..

[16] Vlăduțu D.E., Ionescu M., Mercuț R., Noveri L., Lăzărescu G., Popescu S.M., Scrieciu M., Manolea H.O., Iacov Crăițoiu M.M., Ionescu A.G. (2022). Ecological Momentary Assessment of Masseter Muscle Activity in Patients with Bruxism. Int. J. Environ. Res. Public Health.

[17] Viola C., Muñoz-Corcuera M., Antoranz-Pereda A., Casañas E., Navarrete N. (2024). Time assessment for final restoration of endodontically treated teeth in a university clinic setting: An observational study. Saudi Dent. J..

[18] Maravić T., Comba A., Mazzitelli C., Bartoletti L., Balla I., di Pietro E., Josić U., Generali L., Vasiljević D., Blažić L. (2022). Finite element and in vitro study on biomechanical behavior of endodontically treated premolars restored with direct or indirect composite restorations. Sci. Rep..

[19] Upadhyaya V., Bhargava A., Parkash H., Chittaranjan B., Kumar V. (2016). A finite element study of teeth restored with post and core: Effect of design, material, and ferrule. Dent. Res. J..

[20] Toksavul S., Zor M., Toman M., Güngör M.A., Nergiz I., Artunç C. (2006). Analysis of dentinal stress distribution of maxillary central incisors subjected to various post-and-core applications. Oper. Dent..

[21] Sharafeddin F., Alavi A.A., Zare S. (2014). Fracture resistance of structurally compromised premolar roots restored with single and accessory glass or quartz fiber posts. Dent. Res. J..

[22] Alshabib A., Abid Althaqafi K., AlMoharib H.S., Mirah M., AlFawaz Y.F., Algamaiah H. (2023). Dental Fiber-Post Systems: An In-Depth Review of Their Evolution, Current Practice and Future Directions. Bioengineering.

[23] Nahar R., Mishra S.K., Chowdhary R. (2020). Evaluation of stress distribution in an endodontically treated tooth restored with four different post systems and two different crowns—A finite element analysis. J. Oral Biol. Craniofac. Res..

[24] Oladapo B., Zahedi A., Vahidnia F., Ikumapayi O., Farooq M. (2018). Three-dimensional finite element analysis of a porcelain crowned tooth. Beni-Suef Univ. J. Basic Appl. Sci..

[25] Lin J., Lin Z., Zheng Z. (2020). Effect of different restorative crown design and materials on stress distribution in endodontically treated molars: A finite element analysis study. BMC Oral Health.

[26] Silva N.R., Castro C.G., Santos-Filho P.C., Silva G.R., Campos R.E., Soares P.V. (2009). Influence of different post design and composition on stress distribution in maxillary central incisor: Finite element analysis. Indian J. Dent. Res..

[27] Stănuși A.Ș., Popa D.L., Ionescu M., Cumpătă C.N., Petrescu G.S., Ţuculină M.J., Dăguci C., Diaconu O.A., Gheorghiță L.M., Stănuşi A. (2023). Analysis of Temperatures Generated during Conventional Laser Irradiation of Root Canals-A Finite Element Study. Diagnostics.

[28] Bessone L., Fernandez B.E. (2010). Evaluation of different post systems: Finite element method. Int. J. Odontostomat..

[29] Pierrisnard L., Bohin F., Renault P., Barquins M. (2002). Coronoradicular reconstruction of pulpless teeth: A mechanical study using finite element analysis. J. Prosthet. Dent..

[30] Boțilă M.R., Popa D.L., Mercuț R., Iacov-Crăițoiu M.M., Scrieciu M., Popescu S.M., Mercuț V. (2024). A Finite Element Method Study of Stress Distribution in Dental Hard Tissues: Impact of Access Cavity Design and Restoration Material. Bioengineering.

[31] https://www.3dsystems.com.

[32] https://www.solidworks.com.

[33] https://www.ansys.com.

[34] Maksay S.T., Bistrian D.A. (2008). Introducere în Metoda Elementelor Finite.

[35] Mannocci F., Cowie J. (2014). Restoration of endodontically treated teeth. Br. Dent. J..

[36] McComb D. (2008). Restoration of the Endodontically Treated Tooth.

[37] Staicu A.N., Țuculină M.J., Cumpătă C.N., Rîcă A.M., Beznă M.C., Popa D.L., Popescu A.D., Diaconu O.A. (2024). A Finite Element Method Study on a Simulation of the Thermal Behaviour of Four Methods for the Restoration of Class II Cavities. J. Funct. Biomater..

[38] Petrescu S.M., Rauten A.M., Popescu M., Popescu M.R., Popa D.L., Ilie D., Duță A., Răcilă L.D., Vintilă D.D., Buciu G. (2024). Assessment of Thermal Influence on an Orthodontic System by Means of the Finite Element Method. Bioengineering.

[39] Popescu A.D., Popa D.L., Nicola A.G., Dascălu I.T., Petcu C., Tircă T., Tuculina M.J., Mocanu H., Staicu A.N., Gheorghiță L.M. (2022). Post Placement and Restoration of Endodontically Treated Canines: A Finite Element Analysis Study. Int. J. Environ. Res. Public Health.

[40] Zarow M., Vadini M., Chojnacka-Brozek A., Szczeklik K., Milewski G., Biferi V., D’Arcangelo C., De Angelis F. (2020). Effect of Fiber Posts on Stress Distribution of Endodontically Treated Upper Premolars: Finite Element Analysis. Nanomaterials.

[41] Benazzi S., Nguyen H.N., Kullmer O., Kupczik K. (2016). Dynamic Modelling of Tooth Deformation Using Occlusal Kinematics and Finite Element Analysis. PLoS ONE.

[42] Cicciù M., Cervino G., Bramanti E., Lauritano F., Lo Gudice G., Scappaticci L., Rapparini A., Guglielmino E., Risitano G. (2015). FEM Analysis of Mandibular Prosthetic Overdenture Supported by Dental Implants: Evaluation of Different Retention Methods. Comput. Math. Methods Med..

[43] Keulemans F., Shinya A., Lassila L.V., Vallittu P.K., Kleverlaan C.J., Feilzer A.J., De Moor R.J. (2015). Three-dimensional finite element analysis of anterior two-unit cantilever resin-bonded fixed dental prostheses. Sci. World J..

[44] Hsu M.L., Chang C.L. (2010). Application of Finite Element Analysis in Dentistry.

[45] Yoshimi H., Sasaguri K., Tamaki K., Sato S. (2009). Identification of the occurrence and pattern of masseter muscle activities during sleep using EMG and accelerometer systems. Head Face Med..

[46] Pradeep D., Chandrasekharan N., Aswathy K., Lekshmy A.R. (2023). Flexural Strength is a Critical Property of Dental Materials-An Overview. Acta Sci. Dent. Scienecs.

[47] Mitov G., Dörr M., Nothdurft F.P., Draenert F., Pospiech P.R. (2015). Post-endodontic treatment of incisors and premolars among dental practitioners in Saarland: An interactive Web-based survey. Clin. Oral Investig..

[48] O’Sullivan M. (2005). Fixed Prosthodontics.

[49] Kharboutly N.A., Allaf M., Kanout S. (2023). Three-Dimensional Finite Element Study of Endodontically Treated Maxillary Central Incisors Restored Using Different Post and Crown Materials. Cureus.

[50] Zhu J., Rong Q., Wang X., Gao X. (2017). Influence of remaining tooth structure and restorative material type on stress distribution in endodontically treated maxillary premolars: A finite element analysis. J. Prosthet. Dent..

[51] Al-Omiri M.K., Rayyan M.R., Abu-Hammad O. (2011). Stress analysis of endodontically treated teeth restored with post-retained crowns: A finite element analysis study. J. Am. Dent. Assoc..

[52] Mergulhão V.A., de Mendonça L.S., de Albuquerque M.S., Braz R. (2019). Fracture Resistance of Endodontically Treated Maxillary Premolars Restored with Different Methods. Oper. Dent..

[53] Abduljawad M., Samran A., Kadour J., Karzoun W., Kern M. (2017). Effect of fiber posts on the fracture resistance of maxillary central incisors with Class III restorations: An in vitro study. J. Prosthet. Dent..

[54] Goodacre C.J., Baba N.Z., Ingle J.I., Bakland L.K. (2008). Restoration of Endodontically Treated Teeth. Endodontics.

[55] Shala K., Tmava-Dragusha A., Dula L., Pustina-Krasniqi T., Bicaj T., Ahmedi E., Lila Z. (2018). Evaluation of Maximum Bite Force in Patients with Complete Dentures. Open Access Maced. J. Med. Sci..

[56] Petcu C.M., Niţoi D., Mercuţ V., Tuculină M.J., Iliescu A.A., Croitoru C.I., Diaconu O.A., Iliescu M.G., Gheorghiţă L.M., Iliescu A. (2013). Masticatory tensile developed in upper anterior teeth with chronic apical periodontitis. A finite-element analysis study. Rom. J. Morphol. Embryol..

[57] Wu M.K., van der Sluis L.W., Wesselink P.R. (2004). Comparison of mandibular premolars and canines with respect to their resistance to vertical root fracture. J. Dent..

[58] Lindhe J., Karring T., Araújo M. (2003). Anatomy of the periodontium. Clinical Periodontology and Implant Dentistry.

[59] Walton R.E. (2002). Longitudinal tooth fractures. Principles and Practice of Endodontics.

[60] Hargreaves C. (2020). Cohen’s Pathways of the Pulp.

[61] Schwartz R.S., Jordan R. (2004). Restoration of Endodontically Treated Teeth: The Endodontist’s Perspective.

[62] Faria A.C., Rodrigues R.C., de Almeida Antunes R.P., de Mattos Mda G., Ribeiro R.F. (2011). Endodontically treated teeth: Characteristics and considerations to restore them. J. Prosthodont. Res..

[63] Juloski J., Radovic I., Goracci C., Vulicevic Z.R., Ferrari M. (2012). Ferrule effect: A literature review. J. Endod..

[64] Bacchi A., Dos Santos M.B., Pimentel M.J., Caetano C.R., Sinhoreti M.A., Consani R.L. (2013). Influence of post-thickness and material on the fracture strength of teeth with reduced coronal structure. J. Conserv. Dent..

[65] Eraslan Ö., Eraslan O., Eskitaşcıoğlu G., Belli S. (2011). Conservative restoration of severely damaged endodontically treated premolar teeth: A FEM study. Clin. Oral Investig..

[66] Ceddia M., Lamberti L., Trentadue B. (2024). FEA Comparison of the Mechanical Behavior of Three Dental Crown Materials: Enamel, Ceramic, and Zirconia. Materials.

[67] Uthappa R., Mod D., Kharod P., Pavitra S., Ganiger K., Kharod H. (2015). Comparative evaluation of the metal post and fiber post in the restoration of the endodontically treated teeth. J. Dent. Res. Rev..

[68] Abduljabbar T., Sherfudhin H., AlSaleh S.A., Al-Helal A.A., Al-Orini S.S., Al-Aql N.A. (2012). Fracture resistance of three post and core systems in endodontically treated teeth restored with all-ceramic crowns. King Saud Univ. J. Dent. Sci..

[69] Rodríguez-Cervantes P.J., Sancho-Bru J.L., Barjau-Escribano A., Forner-Navarro L., Pérez-González A., Sánchez-Marín F.T. (2007). Influence of prefabricated post dimensions on restored maxillary central incisors. J. Oral Rehabil..

[70] Bru E., Forner L., Llena C., Almenar A. (2013). Fibre post behaviour prediction factors. A review of the literature. J. Clin. Exp. Dent..

[71] Sethuraman R. (2011). The effect of three post and core systems on the stress distribution in endodontically treated teeth- A two dimensional finite element analysis. J. Adv. Dent. Res..

[72] Pegoretti A., Frambri L., Zappini G., Bianchetti M. (2002). Finite element analysis of a glass fibre reinforced composite endodontic post. Biomaterials.

[73] Badami V., Ketineni H., Pb S., Akarapu S., Mittapalli S.P., Khan A. (2022). Comparative Evaluation of Different Post Materials on Stress Distribution in Endodontically Treated Teeth Using the Finite Element Analysis Method: A Systematic Review. Cureus.

[74] Nokar S., Bahrami M., Mostafavi A.S. (2018). Comparative evaluation of the effect of different post and core materials on stress distribution in radicular dentin by three-dimensional finite element analysis. J. Dent..

[75] Martins M.D., Junqueira R.B., de Carvalho R.F., Lacerda M.F.L.S., Faé D.S., Lemos C.A.A. (2021). Is a fiber post better than a metal post for the restoration of endodontically treated teeth? A systematic review and meta-analysis. J. Dent..

[76] Figueiredo F.E., Martins-Filho P.R., Faria-E-Silva A.L. (2015). Do metal post-retained restorations result in more root fractures than fiber post- retained restorations? A systematic review and meta-analysis. J. Endod..

[77] Dietschi D., Duc O., Krejci I., Sadan A. (2007). Biomechanical considerations for the restoration of endodontically treated teeth: A systematic review of the literature—Part 1. Composition and micro- and macrostructure alterations. Quintessence Int..

[78] Stănuși A., Mercuț V., Scrieciu M., Popescu S.M., Iacov Crăițoiu M.M., Dăguci L., Castravete Ș., Amărăscu M.O. (2020). Analysis of stress generated in the enamel of an upper first premolar A finite element study. Stoma Edu. J..

[79] Jang A.T., Lin J.D., Choi R.M., Choi E.M., Seto M.L., Ryder M.I., Gansky S.A., Curtis D.A., Ho S.P. (2014). Adaptive properties of human cementum and cementum dentin junction with age. J. Mech. Behav. Biomed. Mater..

[80] Jokar H., Rouhi G., Abolfathi N. (2021). The role of PDL-cementum enthesis in protecting PDL under masticatory loading: A finite element investigation. J. Mech. Med. Biol..

